# Evaluation of a rapid, direct sample loading electrochemical AST assay with clinical urine samples

**DOI:** 10.3389/fmicb.2026.1855865

**Published:** 2026-08-12

**Authors:** Oliver Riester, Natalia Kolesnik-Goldmann, Chantal Quiblier, Wilhelm Eugen Andreas Krames, Oliver Nolte, Hans-Peter Deigner

**Affiliations:** 1 Institute of Precision Medicine, Furtwangen University, Villingen-Schwenningen, Germany; 2 Institute of Medical Microbiology, University of Zurich, Zurich, Switzerland; 3 Institute of Organic Chemistry, Martin-Luther University Halle-Wittenberg, Halle (Saale), Germany; 4 Faculty of Science, Eberhard-Karls-University Tuebingen, Tuebingen, Germany; 5 EXIM Department, Fraunhofer Institute IZI (Leipzig), Rostock, Germany

**Keywords:** antimicrobial resistance, antimicrobial susceptibility testing, direct AST, electrochemical test, rapid AST, urine samples

## Abstract

**Introduction:**

Phenotypic antibiotic susceptibility testing (AST) is essential, but conventional culture-based methods require 48–72 h, delaying targeted treatment. Here we report a first small study of a direct electrochemical AST system on clinical urine samples. The study aimed to validate the technology and support further research.

**Methods:**

18 urine and 2 control samples were tested with 3 antibiotics above and below EUCAST breakpoints, resulting in 474 antibiotic-sample combinations grouped as 158 triplets.

**Results:**

Using routine clinical diagnostics as reference method, categorical agreement, sensitivity, and specificity were 87.9% (95% CI, 83.7–91.1%), 0.95 (95% CI, 0.8952–0.9769) and 0.831 (95% CI, 0.7684–0.8786), respectively. The average time to result was 6.0 ± 3.3 h. Its non-species-specific electrochemical principle theoretically allows it to be applied to various sample types and organisms.

**Discussion:**

These results demonstrate that the electrochemical AST platform provides accurate results on the same day directly from patient samples, likely eliminating the need for mandatory pre-culturing.

## 1 Introduction

Phenotypic antimicrobial susceptibility testing (AST) is a cornerstone of infectious disease management, guiding effective antibiotic therapy. With the increasing prevalence of multidrug-resistant pathogens, timely and accurate AST has become critical for both patient outcomes and public health [World Health Organization (WHO), 2025]. Traditionally, AST relies on culture-based methods compatible with all patient specimens, but typically requires 48 to 72 h to yield results (Tabak et al., 2018; Edmiston et al., 2018). The time to result (TTR) and general compatibility of the AST system depends not only on the pathogen under investigation but also on the characteristics of the clinical specimen, such as viscosity, the presence of interfering biomolecules, and the initial bacterial concentration. Bloodstream infections, for example, require initial blood culture incubation (usually 4–24 h, or up to 48 h) (Tabak et al., 2018; MacBrayne et al., 2021) followed by sub-culturing and AST. Urine samples, on the other hand, are less complex and have higher initial bacterial concentrations, thereby offer lower barriers of entry for future non-culture based testing, while the current clinical standard still requires a urine culture prior to susceptibility profiling (Zboromyrska et al., 2016; Zhang et al., 2021). The TTR is particularly important in cases of bloodstream infections and urosepsis when the infection proves resistant to the broad-spectrum antibiotic initially administered, as it can drastically shorten the time to the start of effective antibiotic therapy and thereby reduce the mortality rate. Although most urinary tract infections do not pose a threat to the patient’s life, the rapid initiation of effective antibiotic treatment still offers advantages, such as reducing side effects caused by ineffective antibiotics, making it easier for the physician to decide on a suitable treatment, and curbing the further spread of resistance.

To address the need for faster results, a prerequisite for initiating targeted antibiotic therapy, several rapid AST methods have emerged. For bloodstream infections, platforms such as the Accelerate Pheno™ system (Charnot-Katsikas et al., 2018) and dRAST™ (Halperin et al., 2025) provide susceptibility profiles within 7–9 h after a blood culture becomes positive. For urinary tract infections, systems like BD Phoenix and VITEK 2 offer accelerated workflows, while Couwenberg et al. (2026) note that the PA-100 AST system from Sysmex Astrego is currently the only regulatory-approved phenotypic point-of-care AST device that has been validated for use in uncomplicated urinary tract infections. However, despite these improvements, most existing platforms cannot be loaded directly with patient specimens but require a purified culture or are not intended for use with specimens other than urine.

To overcome existing limitations in fast susceptibility testing, novel AST systems must deliver both rapid and reliable results without preprocessing and in a reasonable time, an essential balance for improving patient outcomes and supporting antibiotic stewardship. Furthermore, it is highly desirable to have a universally applicable test available for many sample types and bacterial species, as is the case with current culture-based workflows. Without such versatility, multiple instruments with heterogeneous workflows would need to be implemented, a scenario that is impractical and costly for direct integration into clinical and physician office settings. In many cases, particularly for more complex sample types or diagnostics, this limitation necessitates referral to external laboratories, introducing delays due to transport logistics and restrictions imposed by laboratory operating hours (Idelevich et al., 2019).

The technology evaluated in this preliminary feasibility study, still in an early stage of development, is based on a miniaturized electrochemical AST platform and still requires manual preparation steps, which currently limits its applicability in primary care settings such as general practitioner clinics where urinary tract infections are predominantly managed. The AST system integrates screen-printed electrodes into a 3D-printed measurement chamber, enabling real-time monitoring of bacterial metabolic activity and integrity under antibiotic exposure. The approach relies on electrochemical readout, allowing direct analysis of complex biological samples without prior isolation or enrichment steps. Key advantages include a simplified workflow, compatibility with different sample matrices, a substantially reduced time to result compared with conventional culture-based AST and its ability to perform AST directly from patient samples or diluted samples, eliminating labor-intensive preparation steps and longer incubation times (Riester et al., 2024). A brief overview of the workflow and test device is given in Supplementary Figure S1. Potential limitations may arise from the dependence on electrochemical signal stability in highly variable sample environments; however, the modular design allows adaptation to different use cases and sample types. The present evaluation demonstrates its suitability for urine samples, as previously shown for spiked serum samples (Riester et al., 2024), highlighting the system’s potential for various sample materials. By simplifying the workflow and reducing the time until results, the method can make a decisive contribution to the early initiation of targeted antimicrobial therapy. The present study demonstrates the applicability directly to urine samples and thus considerably extends the range of application of the method.

## 2 Materials and methods

### 2.1 Device manufacturing

The one chamber devices were fabricated and prepared as previously described (Riester et al., 2024). Briefly, the device geometry was designed using Autodesk Fusion 360 (Autodesk, United States) and processed with the slicer software Ultimaker Cura (v4.6.1, Ultimaker, Netherlands). The devices were fabricated by fused deposition modeling using an Ultimaker 3 3D printer (Ultimaker, Geldermalsen, Netherlands) equipped with a 0.4 mm nozzle and transparent PMMA filament (PMM300XCLR, filamentworld.de, Germany). Printing was performed at 245 °C with a printing speed of 70 mm s^−1^, a layer height of 0.1 mm, and a fan speed of 50%. The printing process was temporarily paused by G-code modification to allow insertion of the ItalSens IS-C screen-printed electrodes with graphite working and counter electrode, and a silver pseudo reference electrode (PalmSens BV, Houten, Netherlands). Commercial ItalSens IS-C electrodes were fixed in place with a minimal amount of superglue applied to the electrode backing material. Following fabrication, the devices were UV-sterilized for 30 min per side under a sterile workbench, sealed with a sterile luer-lock cap and stored in sterile bags until use.

### 2.2 Sample origin

For the purpose of test development and validation, leftover samples from the original samples can be used without ethical approval by diagnostic laboratories. Only urine from patients who had given general consent were used. The Institute of Medical Microbiology (IMM) has an institutional data governance board (DGB) in place, which checks for necessity of ethical approval and GC.

Eighteen anonymized monomicrobial urine samples obtained from secondary and tertiary care hospitals for routine diagnostics at the IMM, University of Zurich were selected for this study. Urine specimens were collected in BD Vacutainer tubes (BD, Franklin Lakes, NJ, USA) containing sodium borate/formate preservative for stabilization of the bacterial cell counts for up to 48 h at room temperatures. Following the routine procedure, the specimens were stored at 4 °C. Preceding urine culture was done by inoculating 10 μL of urine to culture plates (limit of analytical detection therefore 10^3^ CFU/mL). Only plates with pure growth (i.e., monomicrobial growth) of *Escherichia coli* were selected for subsequent experiments. Monomicrobial growth is defined as a pure culture of one culture morphology without any other colony type. For better discrimination, chromogenic agars are used, which support the color-specific growth of species- or higher taxonomic groups (Oxoid Brilliance UTI agar, Thermofisher). Each sample contained at least 10^5^ CFU/mL of *E. coli*. Two quality control strains were included: *E. coli* ATCC 25922 [American Type Culture Collection (ATCC), 2025a] and *Klebsiella pneumoniae* ATCC 700603 [American Type Culture Collection (ATCC), 2025b]. For sterile urine, culture-negative urine of a healthy person was filtered through a 0.22 μm polyethersulfone (PES) filter membrane (TPP, Trasadingen, Switzerland). Based on the reference method, the patient samples comprised 11 resistant and 7 susceptible samples for ampicillin, 7 resistant and 11 susceptible samples for ciprofloxacin, and 1 resistant and 17 susceptible samples for nitrofurantoin.

### 2.3 Microbiological reference method

As reference, EUCAST-standardized disk diffusion served as the routine phenotypic comparator for AST (Matuschek et al., 2014). Inhibition zones were read using the SIRScan instrument (i2a, Montpellier, France) and zone diameters were interpreted into the categories susceptible (S) and resistant (R) according to EUCAST criteria. Ref.: Antimicrobial susceptibility testing EUCAST disk diffusion method, version 13, January 2025.^1^

### 2.4 Sample preparation and electrochemical measurement

For the electrochemical assay, urine samples were prepared by the same laboratory technician, with minor deviations from the method described in Riester et al. (2024). Briefly, stored urine samples without preculture were diluted 1:4 in Mueller-Hinton broth, NutriSelect Plus (Merck Millipore, Burlington, MA, USA) and supplemented with 0.2 mM Resazurin (Thermo Fisher Scientific, Geel, Belgium). For the electrochemical measurement, the 24 prepared devices were each loaded in technical triplicate with the corresponding samples. These included three blank controls containing sterile urine (negative control), three urine samples without antibiotics (positive control), and three urine samples each spiked with the following antibiotics, typically tested in case of *E. coli* UTI: ampicillin, 6 mg/L and 12 mg/L (breakpoint: S ≤ 8 mg/L, R > 8 mg/L); ciprofloxacin, 0.1875 mg/L and 0.75 mg/L (breakpoint: S ≤ 0.25 mg/L, R > 0.25 mg/L, non-meningitis breakpoint); nitrofurantoin, 48 mg/L and 96 mg/L (breakpoint: S ≤ 64 mg/L, R > 64 mg/L). All breakpoints refer to the EUCAST version 15.0, valid as of January 1st 2025 (see text footnote 1). After filling, the devices were inserted into the device holder and connected to the PalmSens4 potentiostat (PalmSens BV, Houten, Netherlands). The connected devices were placed inside an incubator at 37 °C and the parallel measurement was performed using the Software PSTrace (Version 5.11.828, PalmSens BV, Netherlands). The measurements were performed every 10 min over 20 h, where the amount of metabolized resazurin was evaluated with differential pulsed voltammetry (DPV) (*E*_begin_ = −0.8 V; *E*_end_ = 0.0 V; *E*_pulse_ = 0.05 V; *t*_pulse_ = 0.01 s; Scan rate = 0.05 V/s) and the electrochemical impedance spectroscopy (EIS) measurement protocol was performed at frequencies of 10^5^, 10^4^, 10^3^, 10^2^, 10, and 1 Hz with an *E*_dc_ = 0.3 V and *E*_ac_ = 0.01 V.

### 2.5 Data analysis and evaluation

Electrochemical measurement data [EIS: impedance (Z); DPV: current (I) and potential (U)] were exported from PSTrace software for further data management and evaluation using python script version 3.12.0. Data analysis was performed in a single-blinded manner, whereby the analyzer had no knowledge of the identity of the samples during data processing and interpretation. Samples were analyzed for bacterial growth and compared to the control groups (sample without bacteria and sterile sample). Analysis was performed by changepoint detection, as previously described by Riester et al. (2024), without further optimization for urine samples. Briefly, changepoints in the data were detected using the Pruned Exact Linear Time (PELT) algorithm from the ruptures package version 1.1.7 (Truong et al., 2020). Potential (U) was analyzed directly without prior adjustment, whereas for current (I) and impedance (Z), only values obtained after reaching electrochemical equilibrium, as described by Dalheim and Steen (2020), were included in the analysis. Furthermore, changepoint detection for current measurements was performed on the first derivative of I. Each detected changepoint was subsequently evaluated for significant differences relative to the corresponding control measurements without bacterial growth using the SciPy package version 1.10.1 (Virtanen et al., 2020). A changepoint was considered significant when a paired t-test indicated a significant difference (*p* < 0.05) compared with the majority of control samples. For the three electrochemical parameters, a score ranging from 0 to 3 was calculated. Each parameter contributed one point if a significant changepoint was detected at the analysis timepoint or within the preceding hour, with the contribution of each parameter limited to a maximum of one point. A total score of ≥2 was interpreted as bacterial growth. Samples were analyzed independently for each replicate (CP) and additionally as groups of triplicates (CP-grp), where bacterial growth was considered detected if the majority of replicates within a group indicated growth (2 of 3).

### 2.6 Statistical analysis and comparison with clinical standard procedure

Statistical analysis for comparing the new device with the clinical standard method of disc diffusion was performed according to the “Guidance for Industry and FDA: Class II Special Controls Guidance Document: Antimicrobial Susceptibility Test (AST) Systems” (U.S. Department of Health and Human Services Food and Drug Administration Center for Devices and Radiological Health, 2009). Since our study design did not include enough concentrations to evaluate the accurate minimum inhibitory concentration (MIC), the essential agreement (EA) was not evaluated, instead we evaluated the categorical agreement (CA), which compares the interpretive results of the new device under evaluation to the standard reference method. Therefore, we differentiated four categories for comparison: (1) isolates classified as susceptible by the reference method with antibiotic concentrations below the breakpoint (susceptible, low exposure; S^−^), (2) isolates classified as susceptible with concentrations above the breakpoint (susceptible, increased exposure; S^+^), (3) isolates classified as resistant with concentrations below the breakpoint (resistant, low exposure; R^−^), and (4) isolates classified as resistant with concentrations above the breakpoint (resistant, increased exposure; R^+^). All antibiotic - sample combinations that were above the EUCAST—clinical breakpoint according to the standard reference method (R^+^ and S^+^) were categorized as evaluable and included in the evaluation. Antibiotic–sample combinations below the EUCAST clinical breakpoint according to the standard reference method (R^−^ and S^−^) were categorized as non-evaluable and excluded from the primary evaluation. However, these combinations were intentionally included in the experimental design to enable a comprehensive assessment of test performance across clinically relevant and sub-inhibitory concentration ranges and to support future applications involving multiple antibiotic concentrations per active compound. The 95% confidence intervals (CI) were calculated with GraphPad Prism 8 (GraphPad Software, USA) using the method of Wilson/Brown.

## 3 Results

### 3.1 Performance

A total of 18 clinical samples and 2 artificially spiked control-samples were analyzed, resulting in 474 antibiotic—sample combinations tested for concentrations above and below EUCAST breakpoints. Each combination was assessed on three independent devices, yielding individual measurements (CP; *n* = 474), that were also analyzed as in their triplicates (CP-grp; *n* = 158). For reasons of clarity, individual antibiotic-sample combinations are referred to subsequently as combinations and triplicates of antibiotic-sample combinations are referred to as triplicate-combinations. Based on EUCAST criteria, 180 of the 474 combinations were categorized as resistant (R^+^ and R^−^), while 177 were susceptible (S^+^) above breakpoint, and 117 were susceptible (S^−^) below breakpoint. Combinations tested below breakpoint were considered not evaluable (117 S^−^ and 60 R^−^) to avoid potential bias introduced under these conditions, as growth may be detected (indicating resistance) even when the organism is actually susceptible, but the tested concentration is insufficient to inhibit growth. Thereby mentioned CA and subsequentially combinations and triplicate-combinations relate to only evaluable samples (CA including all samples is listed in Table 1). In this study, only CA is reported, as the electrochemical AST system tested a limited number of antibiotic concentrations per sample, which did not allow precise determination of MIC values required for calculating EA.

**Table 1:** Performance of the electrochemical AST system against microbiological reference method (disc diffusion).

Evaluation method	CP	CP-grp
Total tested	474	158
No. CA	365	128
% CA (95% CI)	77.0 (73.0–80.6)	81.0 (77.2–84.3)
Total evaluable	297	99
No. CA of evaluable	248	87
% CA of evaluable (95% CI)	83.5 (78.9–87.3)	87.9 (83.7–91.1)
No. R^+^ and [No. growth detected, controls]	60 [60]	20 [20]
No. VME (%; 95% CI)	10 (8.3; 4.6–14.7)	2 (5.0; 0.9–16.5)
No. ME (%; 95% CI)	39 (22.0; 16.6–28.7)	10 (16.9; 9.5–28.5)
Sensitivity (95% CI)	0.917 (0.892–0.976)	0.950 (0.895–0.977)
Specificity (95% CI)	0.780 (0.713–0.834)	0.831 (0.768–0.879)
Positive predictive value (95% CI)	0.738 (0.662–0.802)	0.792 (0.718–0.850)
Negative predictive value (95% CI)	0.932 (0.912–0.981)	0.961 (0.917–0.982)

For CP analysis, 248 of 297 combinations showed categorical agreement with DD, corresponding to an CA of 83.5% (95% CI: 78.9–87.3%). Within this set, 60 resistant combinations and 60 controls with growth were identified, yielding 10 (8.3% (10/120), 95% CI: 4.6–14.7%) very major errors (VMEs; false susceptibility) and 39 (22.0% (39/177), 95% CI: 16.6–28.7%) major errors (MEs; false resistance). Diagnostic performance metrics were calculated accordingly: overall sensitivity was 0.917 (95% CI: 0.892–0.976), specificity 0.780 (95% CI: 0.713–0.834), positive predictive value 0.738 (95% CI: 0.662–0.802), and negative predictive value 0.932 (95% CI: 0.912–0.981).

CP-grp demonstrated higher concordance. Of the 99 triplicate-combinations, 87 (87.9%, 95% CI: 83.7–91.1%) were in categorical agreement with DD. In this set, 20 resistant triplicate-combinations and 20 controls with growth were identified, with two (5.0% (2/40), 95% CI: 0.9–16.5%) VMEs and 10 (16.9% (10/59), 95% CI: 9.5–28.5%) MEs. Diagnostic performance reached a sensitivity of 0.950 (95% CI: 0.895–0.977), specificity of 0.831 (95% CI: 0.768–0.879), positive predictive value of 0.792 (95% CI: 0.718–0.850), and negative predictive value of 0.961 (95% CI: 0.917–0.982).

VMEs were markedly reduced in the CP-grp analysis compared to the CP analysis. Therefore, all subsequent analyses are presented using the CP-grp method, while additional CP data are provided in Supplementary materials. Table 1 provides a comparative overview, illustrating that evaluation of grouped triplicates (CP-grp) consistently yielded higher performance compared to singlets (CP). Supplementary Table 1 shows results of the electrochemical test (CP-grp) and DD for all tested samples.

Further stratification by antibiotic class revealed heterogeneous performance profiles of the electrochemical AST system, see Table 2 (for additional performance metrics see Supplementary Table 2, for CP data see Supplementary Table 3 and for adapted CP-grp data, where all replicates have to be positive for growth detection, see Supplementary Table 4). Across all evaluable triplicate-combinations, only two VMEs were observed: one in the ampicillin group and one in the nitrofurantoin group. No VMEs were recorded for ciprofloxacin.

**Table 2:** Performance of the electrochemical AST system for each triplicate-combination evaluated with the CP-grp method.

Category	Total Tested	No. CA	% CA (95% CI)	Total evaluable	No. CA of evaluable	% CA of evaluable (95% CI)	No. R^+^ and [No. growth detected, controls]	No. VME (%; 95% CI)	No. ME (%; 95% CI)
Overall samples and controls	158	128	81.0 (77.2–84.3)	99	87	87.9 (83.7–91.1)	20 [20]	2 (5.0; 0.9–16.5)	10 (16.9; 9.5–28.5)
Overall, only samples	118	88	74.6 (69.8–78.8)	59	47	79.7 (73.1–84.9)	20 [0]	2 (10.0; 1.8–30.1)	10 (25.6; 14.6–41.1)
Samples									
Ampicillin	40	27	67.5 (58.7–75.2)	20	14	70.0 (57.5–80.1)	12	1 (8.3; 0.4–35.4)	5 (62.5; 30.6–86.3)
Ciprofloxacin	40	27	67.5 (58.7–75.2)	20	15	75.0 (62.8–84.2)	7	0 (0.0; 0.0–35.4)	5 (38.5; 17.7–64.5)
Nitro-furantoin	38	34	89.5 (82.5–93.9)	19	18	94.7 (85.6–98.6)	1	1 (100.0; 5.1–100.0)	0 (0.0; 0.0–17.6)
Controls									
No antibiotic (sterile urine)	20	20	100.0 (94.0–100.0)	20	20	100.0 (94.0–100.0)	[0]	0 (NA)	0 (0.0; 0.0–16.1)
No antibiotic (patient sample)	20	20	100.0 (94.0–100.0)	20	20	100.0 (94.0–100.0)	[20]	0 (0.0; 0.0–16.1)	0 (NA)

By contrast, the majority of discrepancies with the reference method were classified as MEs. These were most frequent in the ampicillin (62.5% (5/8), 95% CI: 30.6–86.3%) and ciprofloxacin (38.5% (5/13), 95% CI: 17.7–64.5%) groups, which each contained 5 MEs, while nitrofurantoin showed no (0.0% (0/18), 95% CI: 0.0–17.6%) MEs in evaluable samples.

This error distribution was reflected by CA: the highest agreement was shown by nitrofurantoin (94.7%, 95% CI: 85.6–98.6%), while lower agreement was achieved by ampicillin and ciprofloxacin (70.0%, 95% CI: 57.5–80.1% and 75.0%, 95% CI: 62.8–84.2%, respectively). Control conditions, including sterile urine samples and sample without antibiotics, demonstrated 100% CA, confirming the robustness of basic assay performance.

### 3.2 Time to result

**Figure 1:**
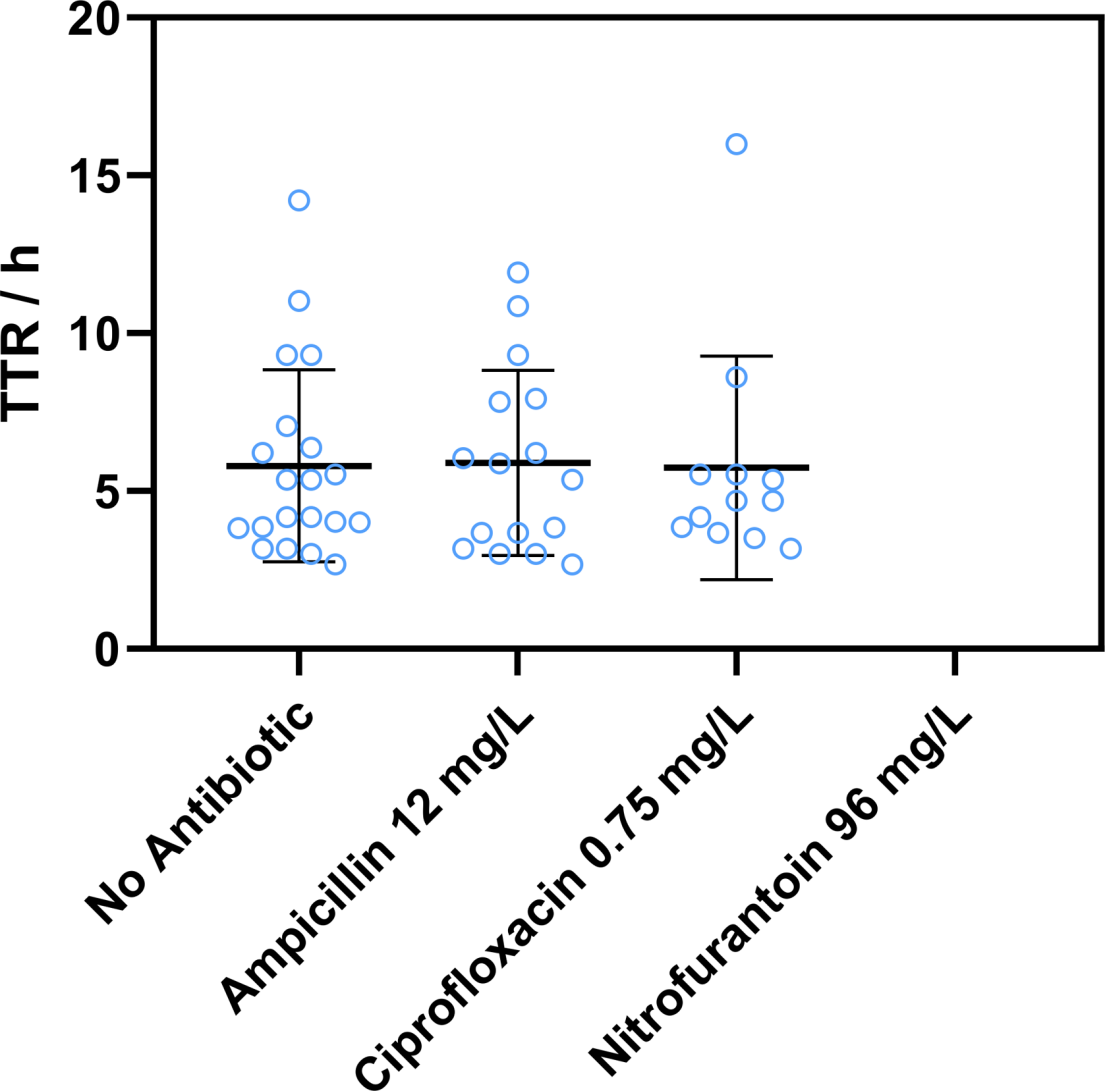
Time to result (TTR), when growth was detected for each antimicrobial on all evaluable tests with the electrochemical assay. Values are shown as mean ± SD in addition to single datapoints (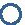) evaluated by CP-grp method. The number of samples, including the control strains, in which growth was observed was 20, 16, and 12, respectively, for the “no antibiotics,” “ampicillin,” and “ciprofloxacin” groups. For Nitrofurantoin, no growth was observed with the CP-grp analysis across all samples, as only one sample was resistant, which is classified as a VME. Adapted from (Riester et al., 2024) with permission under CC BY 4.0.

The average TTR using the CP-grp analysis method was 6.03 ± 3.32 h, see Figure 1. Observed TTR showed no substantial difference when compared between antibiotic groups, as the ampicillin and ciprofloxacin groups showed 5.90 ± 2.84 h and 5.73 ± 3.39 h, respectively. In comparison, for antibiotic-free controls, growth was detected on average within 5.79 ± 2.96 h. For nitrofurantoin, no TTR could be observed for the concentration of 96 mg/L, as no sample with growth was detected using the CP-grp method. These data indicate that the electrochemical AST system generally provides results for urine samples within a clinically relevant time frame, and that the TTR is only slightly influenced by the antibiotic tested and the corresponding inhibitory effects on bacterial growth. Additional data using the CP-grp and CP method are shown in Supplementary Figure S2.

## 4 Discussion

When comparing our AST system with the study by Cardoso et al. (2025), which examined currently established automated systems such as the BD Phoenix system, showing CAs ranging from 68% for cefepime to 100% for levofloxacin, with a mean CA of 79.3%, and the VITEK-2 system, showing CAs ranging from 81% for ciprofloxacin to 100% for four of the antibiotics tested, with a mean CA of 91.2%, the test system described here showed CAs ranging from 70% for ampicillin to 94.7% for nitrofurantoin, with a mean CA of 87.9%. It should be noted that different antibiotics and sample numbers were tested, which may influence the results. These data suggest that our electrochemical AST system achieves performance levels, regarding CA, close to those of widely used routine platforms while retaining the advantage of directly analyzing clinical samples without prior culture.

Nevertheless, concerns remain about the accuracy of widely used semi-automated testing platforms, depending on the origin of the specimen. In a multicenter, retrospective study by Bartoletti et al. (2022) involving 366 patients with monomicrobial bloodstream infections caused by carbapenem-resistant Enterobacterales, semi-automated systems (VITEK 2 or MicroScan 96 plus Walkaway System) showed variable rates of VMEs (highest: 14% for fosfomycin) and MEs (highest: 34% for tigecycline) when compared with reference methods. These inaccuracies led to the prescription or confirmation of ineffective therapy in 7% of cases, which was significantly associated with increased 30-day mortality. For urinary tract infections, a study (Angaali et al., 2018) evaluating the VITEK 2 system screened 844 turbid urine samples. Regarding AST, the system showed a complete agreement rate of 94.3%, with a low incidence of VMEs (0.5%), MEs (2.2%), and minor errors (3%). These findings highlight the generally high reliability of rapid AST systems for urinary tract infections while emphasizing the need for assessment of diagnostic accuracy across sample types.

It has to be mentioned, that VMEs are particularly important because they pose the greatest clinical risk due to their false-positive results. The low absolute number of VMEs of our test system (one in the ampicillin group and one in the nitrofurantoin group) underscores the potential reliability of the platform, although the nitrofurantoin case highlights the limitations of drawing firm conclusions for antibiotics with very few resistant isolates represented in the dataset. In addition, differences in agreement between individual antibiotics may reflect both biological and technical factors. Different antimicrobial classes induce distinct physiological responses and growth dynamics, which may affect the timing and magnitude of electrochemical signals detected by the assay. Furthermore, the current analysis was performed without antibiotic-specific optimization, and the relatively small number of isolates per antibiotic limits the ability to draw robust conclusions regarding compound-specific performance. The observed error rates of 5% VMEs and 16.9% MEs (see Table 1) need further improvement to be comparable to the previous mentioned systems and are likely influenced by the early developmental stage of the assay and the relatively small sample size, which can lead to instability in performance estimates, particularly for antibiotic groups with few resistant isolates. One limitation of this study is that the electrochemical AST system was evaluated against a single reference method, namely an established AST platform that is routinely used in clinical diagnostics. While this approach allows for an assessment of agreement with current standard practice, it does not permit conclusions regarding the absolute clinical superiority of either method. Divergent results may be attributable to limitations of the system under investigation, the reference method, or both. Therefore, the design of this comparative study cannot fully assess the clinical significance of the electrochemical AST test. Future studies conducted during a later stage of the system’s development should include blinded, outcome-oriented evaluations or prospective intervention studies. One of these scenarios may be heteroresistant phenotypes, where a sub population shows an increased resistance, which is often indicated as susceptible by standard methods, as the bacterial concentration of the sub population is too low for detection (Casapao et al., 2013; Heyman et al., 2025). Regarding the prevalence of these heteroresistances, a study by Abbott et al. (2025) examined 291 index isolates from the piperacillin/tazobactam-31 arm of the Phase 3 clinical trial ALLIUM for the treatment of Gram-negative pathogens causing complicated urinary tract infections and found that 11.5% of patients were infected with heteroresistant subpopulations. In our study, testing with the disc diffusion reference method did not reveal any heteroresistant phenotypes among the strains examined, and given the prevalence reported by Abbott et al., it is unlikely that heteroresistant subpopulations influenced the present study, even though these prevalences may vary depending on the pathogen and observed antibiotics. However, given the methodological limitations described above, their presence cannot be definitively excluded.

The aim of this study was to demonstrate the compatibility of the test with clinical urine samples, in addition to previous studies on artificially spiked plasma samples (Riester et al., 2024), as a first step towards proving the tests universal applicability. Unlike many existing rapid systems, which are limited to specific sample types, this platform therefore promises broader use, including the direct testing of blood and other complex materials. Non-evaluable antibiotic-sample combinations were excluded from the primary analysis due to potential bias under the tested antibiotic concentration below the EUCAST breakpoint. To ensure transparency, all test results are provided in Supplementary Table 1.

In addition to accuracy, TTR is a clinical key parameter. The average TTR was 6.03 ± 3.32 h for the CP-grp analysis. As initially stated, currently, the only regulatory-approved phenotypic point-of-care AST system is the PA-100 (Sysmex Astrego), which has been validated for uncomplicated urinary tract infections, highlighting the limited availability of true rapid POC AST solutions. Although the AST time of our electrochemical test for urine samples may be longer than some new AST rapid test platforms requiring between 30 and 90 min (Baltekin et al., 2017; Schoepp et al., 2017; Jiang et al., 2023), it should be noted that urine samples are the sample material with the highest bacterial concentration, so no pre-culture is required for some tests. However, these rapid platforms are typically restricted to specific organisms or limited sample types and therefore cannot yet provide fully universal AST across clinically relevant specimen types. The test by Baltekin et al. (2017), for example, performed well specifically for *E. coli*, but not for *K. pneumoniae* or *Staphylococcus saprophyticus*, highlighting its species-specific limitations. According to the manufacturer, these challenges should be addressed in the PA-100 system from Sysmex. In contrast, the aim of the electrochemical AST platform is to provide a broadly applicable system capable of processing multiple specimen types, which may increase overall clinical flexibility and support future point-of-care deployment through reduced infrastructural requirements. In community-acquired uncomplicated UTIs, currently empirical therapy is typically initiated based on guidelines (Krinner et al., 2024). In the future, faster AST results may reduce follow-up visits and enable earlier therapy adjustment, thereby improving outpatient management and further resistance spread, in which regard the presented test would need further improvement for shorter TTR of UTIs. However, since the broader goal of the AST system is to be universally applicable to multiple infectious syndromes, the test should also be able to be loaded directly with blood samples (second highest annual mortality rate among infectious syndromes worldwide) (Murray et al., 2022), which usually has the lowest bacterial concentrations of sample types, as low as 1–100 CFU/mL or even as low as 0.04 CFU/mL (Loonen et al., 2014). As a result, conventional and also newly developed systems for bloodstream infections always require a pre-culture. Thereby, the TTR takes several days; our electrochemical approach previously achieved a TTR in plasma samples within 5–10 h. For comparison, the AST time (without preculture or sample preparation, which would add at least another 18–24 h (Tabak et al., 2018; MacBrayne et al., 2021) to obtain TTR) for the Reveal, Vitek2 or BD Phoenix systems are 4.6 h (Tibbetts et al., 2022), 9.8 h and 12.1 h (Mittman et al., 2009), respectively. Since symptomatic urinary tract infections show high bacterial concentration of ≥10^5 CFU/mL (Coulthard, 2019), this usually poses no limitation. Previous feasibility tests indicate that with the electrochemical assay, an analytical limit of detection of one bacterium per measurement chamber seems viable, which relates to initial bacterial concentrations of 20 CFU/mL (200 μL chamber size and 4x dilution of sample with medium). Although such low limits are methodologically impressive, their diagnostic value is limited because bacterial populations in clinical infections are typically heterogeneous rather than derived from a single clone (Foxman, 2010). This is why conventional AST uses material from several colonies to better reflect this diversity. The direct loading approach enables a significant time advantage, particularly in the case of serious infections such as bloodstream infections. This makes our approach highly relevant as it is potentially applicable to all types of samples and provides results within one working day. As a result, the number of required test systems in a laboratory can be significantly reduced, allowing smaller laboratories to operate with a single portable device while referring specialized or unclear cases to larger reference laboratories for additional testing.

In terms of analysis methods, alternative approaches, including dynamic time warping or combined analysis strategies were tested, but did not show superior performance compared to changepoint-based assessment at the current stage of development. Nevertheless, alternative assessment approaches could improve the discrimination between resistant and susceptible phenotypes and reduce MEs and VMEs in the future, while AI-assisted evaluation of multidimensional signal data is likely to improve classification accuracy and reduce error rates beyond the level achievable with threshold-based methods.

There are some limitations to this study that need to be addressed in the future. First, the relatively small overall sample size and the limited number of resistant samples for certain antibiotic classes restricted the statistical power and depth of performance analyses. In addition, the proof of concept study focused primarily on *E. coli* as the most common pathogen causing urinary tract infections (Flores-Mireles et al., 2015), and a reference strain of *K. pneumoniae*, which may limit the generalizability of the results to other clinically relevant uropathogens. However, the principle of electrochemical detection is not species-specific, as previously demonstrated for *E. coli*, *S. aureus*, *K. pneumoniae* and *Pseudomonas aeruginosa* (Riester et al., 2024).

Theoretically, polymicrobial analysis should be possible, although only the overall susceptibility of the mixed population could be assessed rather than that of individual species. Since AST results would be available before species identification, testing multiple antibiotic concentrations remains important to maximize the likelihood of effective pathogen inhibition. Polymicrobial cases represent only a minority of bloodstream infections (11.4%) (Pavlaki et al., 2013) and urinary tract infections (25%) (Piatti et al., 2025), but interpretation in urinary diagnostics is challenging. Mixed urine cultures with more than one predominant microbe complicate diagnosis, and while standard guidelines consider more than two isolates at >10,000 CFU/mL as contamination, asymptomatic bacteriuria is common and the presence of an indigenous urinary microbiome challenges the assumption that urine is sterile (Moreland et al., 2025). Direct AST testing of the specimen has certain limitations in cases of polymicrobial infections; however, given the relative rarity and low clinical relevance of polymicrobial urinary tract infections, the benefits of the shortened TTR outweigh these drawbacks.

## 5 Conclusion

In conclusion, the electrochemical AST platform has great potential for rapid and accurate antibiotic susceptibility testing directly from clinical samples. It was shown, that the TTR for urine with 6.0 ± 3.3 h was comparable to established methods and can enable timely clinical decision making. The advantage over existing methods is, that the electrochemical test is also suitable for complex matrices and sample types such as serum, which was shown in previous studies. The direct analysis of urine samples presented here, without prior culture, demonstrates the method’s applicability to samples from other sources, with the goal of demonstrating its potential universal applicability in the future. This would represent a significant advantage over traditional culture-based approaches and also make it attractive for smaller laboratories and resource-limited settings. Future work should focus on expanding the range of pathogens and antibiotics tested, optimizing the assay design for low volume or low bacterial concentration samples, and improve data analysis, for example by integrating AI to fully exploit the multidimensional electrochemical signals. Additional studies will provide comprehensive, clinically actionable AST results to assess potential performance beyond standard reference methods.

## Data Availability

The original contributions presented in the study are included in the article/Supplementary material, further inquiries can be directed to the corresponding author.

## Footnotes

1
www.eucast.org
