# Supplementary material for: Evaluation of a rapid, direct sample loading electrochemical AST assay with clinical urine samples
Source: Front Microbiol. 2026 Aug 12;17:1855865. doi: 10.3389/fmicb.2026.1855865 (PMC13506742; doi:10.3389/fmicb.2026.1855865)
Supplement: Supplementary file 1 [file Table_1.DOCX]

Supplementary Material

**Evaluation of a rapid, direct sample loading electrochemical AST assay with clinical urine samples**

Oliver Riester ^1^, Natalia Kolesnik-Goldmann ^2^, Chantal Quiblier ^2^, Wilhelm Eugen Andreas Krames ^1,3^, Oliver Nolte ^2^, Hans-Peter Deigner ^1,4,5,*^

^1^ Institute of Precision Medicine, Furtwangen University, Jakob-Kienzle-Strasse 17, Villingen-Schwenningen, 78054, Germany

^2^ Institute of Medical Microbiology, University of Zurich, Gloriastrasse 28/30, Zurich, 8006, Switzerland

^3^ Institute of Organic Chemistry, Martin-Luther University, Halle-Wittenberg, 06120, Halle (Saale), Germany

^4^ Faculty of Science, Eberhard-Karls-University Tuebingen, Auf der Morgenstelle 8, Tuebingen, 72076, Germany

^5^ EXIM Department, Fraunhofer Institute IZI (Leipzig), Schillingallee 68, Rostock, 18057, Germany

*** Correspondence:**
Corresponding Author
hans-peter.deigner@hs-furtwangen.de

Keywords: Antimicrobial susceptibility testing, antimicrobial resistance, electrochemical test, direct AST, rapid AST, urine samples.

Abstract

Phenotypic antibiotic susceptibility testing (AST) is essential, but conventional culture-based methods require 48 to 72 hours, delaying targeted treatment. Here we report a first small study of a direct electrochemical AST system on clinical urine samples. The study aimed to validate the technology and support further research. 18 urine and 2 control samples were tested with 3 antibiotics above and below EUCAST breakpoints, resulting in 474 antibiotic-sample combinations grouped as 158 triplets. Using routine clinical diagnostics as reference method, categorical agreement, sensitivity, and specificity were 87.9% (95% CI: 83.7% – 91.1%), 0.95 (95% CI: 0.8952 – 0.9769) and 0.831 (95% CI: 0.7684 – 0.8786), respectively. The average time to result was 6.0 ± 3.3 hours. Its non-species-specific electrochemical principle theoretically allows it to be applied to various sample types and organisms. These results demonstrate that the electrochemical AST platform provides accurate results on the same day directly from patient samples, likely eliminating the need for mandatory pre-culturing.

# 1 Overview about the test device und workflow and future improvements

The methodology described in Riester et al. (Riester et al., 2024) follows a workflow for the phenotypic determination of antibiotic susceptibility, which is schematically illustrated in **Figure S1**: First, the bacterial sample is mixed with culture medium and a redox indicator (resazurin) and incubated with defined concentrations of the antibiotics to be tested. The prepared solutions are then transferred into the 3D-printed single-chamber measuring cell (**Figure S1 A**), which contains an integrated screen-printed electrode and can be directly connected to a potentiostat. At the current stage of development, each antibiotic to be tested must be placed in a separate single-chamber device, and 24 such devices can be tested in parallel. In the future, more parallel tests could be conducted by adding additional multiplexers to the experimental setup, or the devices could be combined into a single multi-chamber cartridge to simplify handling. The system is currently compatible with additive manufacturing and can be fully produced by 3D printing, which is advantageous for rapid prototyping and iterative development, whereas large-scale commercial production is expected to rely on injection molding due to lower costs, higher throughput, and improved manufacturing consistency. The electrochemical analysis is performed in parallel with the incubation using two complementary measurement principles: First, the metabolic activity of the bacteria is measured via the reduction of resazurin to resorufin using differential pulse voltammetry (**Figure S1 B**). Second, changes in cell integrity and surface properties are detected via electrochemical impedance measurements (Figure S1C). Both signals are recorded at defined time intervals, enabling a time-resolved characterization of the bacterial response to antibiotic exposure. The analysis is based on a comparison of signal profiles between antibiotic-containing samples and a control without the active ingredient. While resistant bacteria exhibit metabolic activity and corresponding impedance signatures, susceptible strains show a significantly reduced signal response. The entire process, from sample preparation through measurement to data analysis, is summarized in **Figure S1 D)** and enables phenotypic assessment within a few hours without prior isolation or enrichment steps.

Future assay designs may benefit from processing larger initial sample volumes to ensure that each chamber receives a sufficiently representative number of CFU while maintaining high analytical sensitivity. A potential future strategy will apply multiple electrodes within a single 1 - 2 mL chamber to conserve sample volume, rather than triplicate measurements requiring additional input. Particularly for blood samples, this approach in combination with a short 1 - 2 hours enrichment procedure - that could take place during the initial wetting phase of electrodes - would align the system with clinical blood culture practice even for lowest bacterial concentrations, where 2 - 4 blood culture sets are typically collected, corresponding to approximately 40 - 80 mL of blood in total (distributed across aerobic and anaerobic bottles, typically 10 mL each) (U.S. Centers for Disease Control and Prevention, 2025).

Such an approach may be particularly valuable for sample types that currently require a pre-culture step before conventional AST can be performed, as the overall time to clinically actionable susceptibility results is often dominated by this initial cultivation period rather than the AST itself. While the electrochemical platform is not intended to replace established laboratory AST systems in its current form, particularly as these typically integrate pathogen identification alongside susceptibility testing, it could complement existing workflows by providing earlier susceptibility information in time-critical situations where direct testing is feasible.

**Supplementary Figure S1.** Overview about the test device und workflow. **A)** Image of one chamber test device with integrated electrode, that can be filled and connected to the potentiostat. **B)** General principle of resazurin measurement with differential pulsed voltammetry and **C)** exemplary curve of electrochemical impedance measurement of intact bacteria. **D)** Simplified workflow from sample to analysis. Adapted from (Riester et al., 2024) with permission under CC BY 4.0.

# 2 Additional data

An overview of the interpreted results obtained for the 20 tested samples by disc diffusion as the clinical reference method and the CP-grp method for the electrochemical assay are shown in Supplementary Table 1. Data shown include evaluable and non-evaluable combinations.

**Supplementary Table 1.** Results of Electrochemical AST system and clinical reference method (disc diffusion, DD) by sample. Controls (+: Growth detected, -: No growth detected); R: Resistant, S: Susceptible.

|  | Control (no antibiotic, sterile) | | Control (no antibiotic, patient sample) | | Ampicillin 6mg/ml | | Ampicillin 12mg/ml | | Ciprofloxacin 0.1875 mg/L | | Ciprofloxacin 0.75 mg/L | | Nitrofurantoin 48 mg/L | | Nitrofurantoin 96 mg/L | |
| --- | --- | --- | --- | --- | --- | --- | --- | --- | --- | --- | --- | --- | --- | --- | --- | --- |
| Sample | CP-grp | DD | CP-grp | DD | CP-grp | DD | CP-grp | DD | CP-grp | DD | CP-grp | DD | CP-grp | DD | CP-grp | DD |
| *E. coli* ATCC 25922 | - | - | **+** | **+** | S | S | S | S | S | S | S | S | S | S | S | S |
| *K. pneumoniae* ATCC 700603 | - | - | **+** | **+** | **R** | **R** | **R** | **R** | **R** | S | S | S | S | NA | S | NA |
| Urine 1 | - | - | **+** | **+** | **R** | S | **R** | S | S | S | S | S | S | S | S | S |
| Urine 2 | - | - | **+** | **+** | **R** | **R** | **R** | **R** | **R** | S | S | S | **R** | S | S | S |
| Urine 3 | - | - | **+** | **+** | **R** | S | **R** | S | **R** | S | **R** | S | S | S | S | S |
| Urine 4 | - | - | **+** | **+** | **R** | S | **R** | S | **R** | **R** | **R** | **R** | S | S | S | S |
| Urine 5 | - | - | **+** | **+** | **R** | **R** | **R** | **R** | **R** | S | **R** | S | **R** | S | S | S |
| Urine 6 | - | - | **+** | **+** | **R** | **R** | **R** | **R** | **R** | S | **R** | S | S | S | S | S |
| Urine 7 | - | - | **+** | **+** | **R** | S | **R** | S | **R** | S | S | S | S | S | S | S |
| Urine 8 | - | - | **+** | **+** | **R** | S | S | S | S | S | S | S | **R** | S | S | S |
| Urine 9 | - | - | **+** | **+** | **R** | **R** | S | **R** | **R** | **R** | **R** | **R** | S | S | S | S |
| Urine 10 | - | - | **+** | **+** | **R** | **R** | **R** | **R** | **R** | **R** | **R** | **R** | S | S | S | S |
| Urine 11 | - | - | **+** | **+** | **R** | **R** | **R** | **R** | **R** | **R** | **R** | **R** | S | S | S | S |
| Urine 12 | - | - | **+** | **+** | **R** | **R** | **R** | **R** | S | S | S | S | S | S | S | S |
| Urine 13 | - | - | **+** | **+** | **R** | **R** | **R** | **R** | **R** | **R** | **R** | **R** | S | S | S | S |
| Urine 14 | - | - | **+** | **+** | **R** | S | **R** | S | **R** | **R** | **R** | **R** | S | S | S | S |
| Urine 15 | - | - | **+** | **+** | **R** | **R** | **R** | **R** | **R** | S | **R** | S | **R** | **R** | S | **R** |
| Urine 16 | - | - | **+** | **+** | **R** | **R** | **R** | **R** | **R** | **R** | **R** | **R** | S | S | S | S |
| Urine 17 | - | - | **+** | **+** | **R** | **R** | **R** | **R** | **R** | S | **R** | S | S | S | S | S |
| Urine 18 | - | - | **+** | **+** | **R** | S | S | S | S | S | S | S | S | S | S | S |

**Supplementary Table 2.** Additional performance metrics of the Electrochemical AST system for each antibiotic - sample combination using the CP-grp method evaluating each replicate individually.

|  | **Sensitivity**  (95% CI) | **Specificity**  (95% CI) | **Positive predictive value**  (95% CI) | **Negative predictive value** (95% CI) |
| --- | --- | --- | --- | --- |
| Overall (Samples & Controls) | 0.950  (0.895 – 0.977) | 0.831  (0.768 – 0.879) | 0.792  (0.718 – 0.850) | 0.961  (0.917 – 0.982) |
| Overall (Samples) | 0.900  (0.799 – 0.953) | 0.744  (0.658 – 0.814) | 0.643  (0.536 – 0.737) | 0.936  (0.866 – 0.970) |
| **Samples** |  |  |  |  |
| Ampicillin | 0.917  (0.782 – 0.971) | 0.375  (0.212 – 0.573) | 0.688  (0.547 – 0.801) | 0.750  (0.468 – 0.911) |
| Ciprofloxacin | 1.000  (0.845 – 1.000) | 0.645  (0.459 – 0.751) | 0.583  (0.422 – 0.729) | 1.000  (0.862 – 1.000) |
| Nitrofurantoin | 0.000  (0.000 – 0.562) | 1.000  (0.934 – 1.000) | NA | 0.947  (0.856 – 0.986) |
| **Controls** |  |  |  |  |
| No antibiotic (sterile urine) | NA | 1.000  (0.940 – 1.000) | NA | 1.000  (0.940 – 1.000) |
| No antibiotic  (patient sample) | 1.000  (0.940 – 1.000) | NA | 1.000  (0.940 – 1.000) | NA |

The supplementary Tables 3 and 4 show the data analogous to Table 2, but supplementary Table 3 was analyzed using the CP method rather than the CP-grp method and supplementary Table 4 was analyzed with an adapted version of the CP-grp method, where bacterial growth is indicated by 3 positives of triplicates (3 of 3) instead of the 2 positive of triplicates (2 of 3).

**Supplementary Table 3.** Performance of the Electrochemical AST system for each antibiotic - sample combination using the CP method evaluating each replicate individually.

|  | **Total Tested** | **No. CA** | **% CA**  (95% CI) | **Total Evalu-able** | **No. CA of Evalu-able** | **% CA of Evaluable**  (95% CI) | **No. R+ and [No. Growth detected, Controls]** | **No. VME**  (%; 95% CI) | **No. ME**  (%; 95% CI) |
| --- | --- | --- | --- | --- | --- | --- | --- | --- | --- |
| Overall (Samples & Controls) | 474 | 365 | 77.0  (73.0 – 80.6) | 297 | 248 | 83.5  (78.9 – 87.3) | 60[60] | 10  (8.3; 4.6 – 14.7) | 39  (22.0; 16.6 – 28.7) |
| Overall (Samples) | 354 | 248 | 70.1  (65.1 – 74.6) | 177 | 131 | 74.0  (67.1 – 79.9) | 60[0] | 7  (11.7; 5.8 – 22.2) | 39  (33.3; 25.4 – 42.3) |
| **Samples** |  |  |  |  |  |  |  |  |  |
| Ampi-cillin | 120 | 77 | 64.2  (55.3 – 72.2) | 60 | 40 | 66.7  (54.1 – 77.3) | 36 | 4  (11.1; 4.4 – 25.3) | 16  (66.7; 46.7 – 82.0) |
| Cipro-floxacin | 120 | 80 | 66.7  (57.8 – 74.5) | 60 | 43 | 71.7  (59.2 – 81.5) | 21 | 0  (0.0; 0.0 – 15.5) | 17  (43.6; 29.3 – 59.0) |
| Nitro-furantoin | 114 | 91 | 79.8  (71.5 – 86.2) | 57 | 48 | 84.2  (72.6 – 91.5) | 3 | 3  (100.0; 43.9 – 100.0) | 6  (11.1; 5.2 – 22.2) |
| **Controls** |  |  |  |  |  |  |  |  |  |
| No antibiotic (sterile urine) | 60 | 60 | 100.0  (94.0 –100.0) | 60 | 60 | 100.0  (94.0 – 100.0) | [0] | 0  (NA) | 0  (0.0; 0.0 – 6.0) |
| No antibiotic  (patient sample) | 60 | 57 | 95.0  (86.3 – 98.6) | 60 | 57 | 95.0  (86.3 – 98.6) | [60] | 3  (5.0; 17.5 – 36.7) | 0  (NA) |

**Supplementary Table 4.** Performance of the Electrochemical AST system for each antibiotic - sample combination using an adaption of the CP-grp method where 3 of the triplicates have to be positive for a positive result (3 of 3).

|  | **Total Tested** | **No. CA** | **% CA**  (95% CI) | **Total Evalu-able** | **No. CA of Evalu-able** | **% CA of Evaluable**  (95% CI) | **No. R+ and [No. Growth detected, Controls]** | **No. VME**  (%; 95% CI) | **No. ME**  (%; 95% CI) |
| --- | --- | --- | --- | --- | --- | --- | --- | --- | --- |
| Overall (Samples & Controls) | 158 | 134 | 84.8  (81.3 to 87.8) | 99 | 87 | 87.9  (83.7 to 91.1) | 20[20] | 5  (12.5; 5.5 – 26.1) | 7  (11.9; 5.9 – 22.5) |
| Overall (Samples) | 118 | 88 | 74.6  (77.0 to 85.1) | 59 | 49 | 83.1  (76.8 to 87.9) | 20[0] | 3  (15.0; 5.2 – 36.0) | 7  (17.9; 9.0 – 32.7) |
| **Samples** |  |  |  |  |  |  |  |  |  |
| Ampi-cillin | 40 | 25 | 62.5  (53.6 to 70.6) | 20 | 13 | 65.0  (52.4 to 75.8) | 12 | 2  (16.7; 3.0 – 44.8) | 5  (62.5; 30.6 – 86.3) |
| Cipro-floxacin | 40 | 34 | 85.0  (77.5 to 90.3) | 20 | 18 | 90.0  (79.9 to 95.3) | 7 | 0  (0.0; 0.0 – 35.4) | 2  (15.4; 2.7 – 42.2) |
| Nitro-furantoin | 38 | 37 | 97.4  (92.5 to 99.3) | 19 | 18 | 94.7  (85.6 to 98.6) | 1 | 1  (100.0; 5.1 – 100.0) | 0  (0.0; 0.0 – 17.6) |
| **Controls** |  |  |  |  |  |  |  |  |  |
| No antibiotic (sterile urine) | 20 | 20 | 100.0  (94.0 to 100.0) | 20 | 20 | 100.0  (94.0 to 100.0) | [0] | 0  (NA) | 0  (0.0; 0.0 – 16.1) |
| No antibiotic  (patient sample) | 20 | 18 | 90.0  (79.9 to 95.3) | 20 | 18 | 90.0  (79.9 to 95.3) | [20] | 2  (10.0; 1.8 – 30.1) | 0  (NA) |

Figure S2 shows the data analogous to Figure 1, but includes the evaluation using the CP method in addition to the CP-grp method and includes evaluable and non-evaluable combinations.

**Supplementary Figure S2.** Time to result (TTR), when growth was detected for each antimicrobial on all evaluable and non-evaluable tests with the electrochemical assay. Values are shown as mean ± SD in addition to single datapoints evaluated by CP-grp method (○) and CP method (□).

# 3 References

U.S. Centers for Disease Control and Prevention (2025). Preventing Adult Blood Culture Contamination: A Quality Tool for Clinical Laboratory Professionals. Available at: <https://www.cdc.gov/lab-quality/docs/BCC-Prevention_A-Quality-Tool_CDC.pdf> (Accessed October 13, 2025).

Riester, O., Kaiser, L., Laufer, S., and Deigner, H. (2024). Rapid Phenotypic Antibiotics Susceptibility Analysis by a 3D Printed Prototype. *Advanced Science* 11, 2308806. doi: [10.1002/advs.202308806](https://doi.org/10.1002/advs.202308806)
